# Gynecological Fistulae—Has Anything Changed in the Diagnosis and Treatment over the Last Decade? A Narrative Literature Review

**DOI:** 10.3390/medicina59081455

**Published:** 2023-08-12

**Authors:** Marek Misiak, Michalina Dworak, Małgorzata Wyszomirska, Maria Kurt, Maciej Walędziak, Anna Różańska-Walędziak

**Affiliations:** 1 Interdisciplinary Students Association of Metabolic and Systemic Disease “Salus Aegroti” Faculty of Medicine, Collegium Medicum, Cardinal Stefan Wyszynski University in Warsaw, 01-815 Warszawa, Poland; misiak.m2022@gmail.com (M.M.); michalina.m.dworak@gmail.com (M.D.); m.wyszomirska30@gmail.com (M.W.); mariakurt@wp.pl (M.K.); 2 Department of General, Oncological, Metabolic and Thoracic Surgery, Military Institute of Medicine—National Research Institute, Szaserów 128 St., 04-141 Warsaw, Poland; 3 Department of Human Physiology and Pathophysiology, Faculty of Medicine, Collegium Medicum, Cardinal Stefan Wyszynski University in Warsaw, 01-815 Warszawa, Poland; aniaroza@tlen.pl

**Keywords:** gynecological fistulae, obstetric fistulae, cesarean section, *Schistosoma haematobium*, Crohn’s disease, endometriosis, vesicovaginal fistulae, urethrovaginal fistulae, vesico-uterine fistulae, vaginoplasty, urethrovaginal fistulae, rectovaginal fistulae, ano-vaginal fistulae

## Abstract

Gynecological fistulae are a rare but severe complication of radiation therapy, pelvic surgery, prolonged labor, cesarean deliveries, or inflammatory bowel diseases. A gynecological fistula is an abnormal pathway formed between the urinary and gynecological tract, most commonly located between the urinary bladder and vagina. Vesico-uterine and vesicovaginal fistulae are an important health issue, common in women of reproductive age in developing countries with limited access to obstetrical care. Various surgical techniques have been described for VVF repair, depending on the location, severity, and cause of the fistula and the surgeon’s experience. The purpose of our review was to evaluate the present state of knowledge about the prevalence and treatment of gynecological fistulae. The PubMed scientific database was searched for original articles on the subject of gynecological fistulae that had been published between 2013 and 2023.

## 1. Introduction

A pathological connection between the vagina and other surrounding organs, such as the bladder, rectum, or uterus, is defined as a gynecological fistula. The connection enables urine or feces to enter the vagina, which can lead to leakage and many associated symptoms. Radiation therapy, pelvic surgery, delivery complications, or inflammatory illnesses such as Crohn’s disease are the most common factors responsible for gynecological fistulae [1]. Locally advanced gynecological malignancies, such as cervical cancer, and previous radiation therapy due to pelvic malignancies, are among the causes of an abnormal pathway between the urinary and gynecological tract and are most commonly located between the urinary bladder and vagina. There are also many cases of vesico-uterine fistulae, mostly in younger women with a previous history of pelvic surgical procedures [2].

The most important types of genitourinary fistulae are vesicovaginal, urethrovaginal, vesico-uterine, urethrocutaneous, or combined (multiple) fistulae. Vesicovaginal fistulae (usually secondary to a prolonged second stage of labor) are most common in communities with a high rate of out-of-hospital births. With urbanization and a consequent tendency of births to occur in a hospital setting, there has been an increase in the frequency of this kind of fistulae secondary to medical interventions, such as cesarean sections, hysterectomies, and others, as well as secondary to malignant diseases, caused by the diseases themselves or by the treatment interventions. The latter fistulae are more often of the urethrovaginal or vesico-uterine type [3].

Vesicovaginal fistula (VVF), which is a connection between the bladder and vagina, is the most prevalent type of fistula. In most cases, it is caused by obstetrical and gynecological damage or interventions. VVFs cause persistent urine leakage from the vagina, which immensely deteriorates the patient’s quality of life (QUALY). Gynecological surgery is the most important factor responsible for VVFs in higher-income countries, with the most common cause of VVFs being a bladder injury during a hysterectomy [4].

An endometriotic lesion in the rectum or other part of the larger intestine leads to an inflammation process, tissue damage, and the development of a pathological connection between the bowel and vagina [5]. A connection between the rectum and vagina is called rectovaginal fistula (RVF), which is responsible for such symptoms as feces or urine incontinence, pathological vaginal discharge, recurrent pain, and chronic infections due to bacteria transfer from the intestinal lumen to the vagina. Vesico-uterine fistulae can occur after hysteroscopy, in association with endometriosis, intrauterine device migration, inflammatory bowel disease, or due to urinary bladder tuberculosis [2].

The purpose of this study was to evaluate the existing state of knowledge about different types of gynecological fistulae and the efficacy of their treatment.

## 2. Materials and Methods

The PubMed scientific database was searched for original articles on the subject of gynecological fistulae that had been published between 2013 and 2023. The research was performed by four independent researchers under the supervision of the senior researcher, and the articles were retrieved using Google Scholar. The search terms were as follows: “gynecological fistulae”, “obstetric fistulae”, “vesicovaginal fistulae”, “urethrovaginal fistulae”, “vesico-uterine fistulae”, “vaginoplasty”, “urethrovaginal fistulae”, “rectovaginal fistulae”, and “ano-vaginal fistulae”. The selected literature consists of clinical trials and randomized controlled trials. We divided our review into sections, including diagnosing fistulae, treatment of the fistulae, obstetric fistulae, cesarean sections and fistulae, pregnancy and childbirth after repair of obstetric fistulae, *Schistosoma haematobium* and fistulae, endometriosis and fistulae, Crohn’s disease and fistulae, vaginoplasty and fistulae, and gynecological oncology and fistulae.

## 3. Results

### 3.1. Diagnosing Fistulae

The visualization of fistulae using radiology imaging is of great utility both in diagnosis and therapy planning. Gynecological fistulae can be found using a variety of techniques. Radiological imaging allows identifying both the fistula itself and the possible lesions in the surrounding tissues, such as the bladder, urethra, or rectum. It can also be used in deciding about the optimum treatment plan and the surgery protocol. Ultrasound examination can identify intestinal, bladder, ovarian, or uterus abnormalities, being non-invasive and providing preliminary information [6].

Conventional radiography scans can identify calcifications or other bone alterations that may be related to a fistula in the pelvic or urinary tract, even though it is not typically used as the primary imaging technique for fistula diagnosis. The conventional vaginography exam is a radiographic technique that has been used in diagnosing vaginal fistulae since the 1960s as a simple and accurate method. Contrast is injected into the vagina and images are acquired to detect the possible presence of a fistula. Computer tomography vaginography is a more precise method that allows the evaluation of anatomy and permeability of the fistula and can help the surgeon to plan the optimum surgical approach. The resulting images can show the position and size of the fistula, as well as any alterations in surrounding structures, such as the intestine, bladder, or urethra [7].

Magnetic resonance imaging (MRI) is the method of choice in diagnosing fistulae, as it allows detailed visualization of the pelvic and perineal structures, including the anal sphincter muscles. MRI affords the possibility to distinguish between malignant tissue, post-surgical fibrosis, acute inflammatory changes, or abscesses. Thus, if the patient’s clinical parameters allow it, MRI is the suggested imaging technique to investigate the presence of suspected urethro-, ano-, and recto-vaginal fistulae [8].

### 3.2. Treatment of Fistulae

There are different methods of treatment of VVF, including fulguration, occluding devices, prolonged catheterization, open repair, and laparoscopic or robotic surgery [9,10,11,12]. The typical treatment for gynecological fistulae is the surgical excision of the abnormal connection to reestablish normal function. Laparoscopy is of great advantage when compared to open surgery, including a lower postoperative pain level, smaller incisions, lower blood loss, a shorter hospital stay, and faster recovery. It is associated with a lower incidence of postoperative complications, including infections and improper wound healing [13,14]. Physiotherapy is of great importance in achieving full recovery. In a clinical trial by Castille et al., where the effect of postoperative physiotherapy was compared to a placebo, patients in the control group were much more likely to experience postoperative urine incontinence than patients in the physiotherapy group. Additionally, the physiotherapy group had chances of full postoperative recovery 2.72 times higher than the control group [15].

Healing of a fistula is defined as its closure and the return of continence. In the case of a more complex fistulae, healing is not always successful after the first attempt. Moreover, reoperations may have lower success rates than initial repairs. The results of the operation depend on the degree of scarring and tissue loss, the size and location of the fistula, and the surgeon’s experience. In many countries, access to skilled professionals capable of repairing a fistula remains limited, and very few hospitals are providing the service [16]. Other issues that reduce the chance of a successful surgery and postoperative period are the lack of constant care and long waiting lists for the surgery [17]. Adequate health education and physiotherapy programs improve the likelihood of a successful outcome after the surgical repair of an obstetric fistula [15].

A study by Gebremedhin et al. showed that women with no formal education have been seeking care less often compared to those with a formal education [18]. This may be because women with no formal education are more likely to have a low economic status, tend to be economically dependent on others, and may lack household decision-making power. The low educational status may limit women’s awareness about the existence of treatment for fistulae as well. Adolescent girls are also less likely to seek care for fistula treatment. Only about 74% of women in the study attained complete continence after surgery, which suggests the suboptimal quality of the surgical care or the presence of other contextual factors that may limit surgical closure (e.g., malnutrition) in the region. Facility-based studies conducted in Ethiopia, Rwanda, Nigeria, and Guinea reported complete continence rates to range between 83% and 89% after the surgical closure of fistulae, however, it is difficult to compare figures between different countries due to different surgical approaches.

### 3.3. Obstetric Fistulae

An obstetric fistula is defined as a pathological pathway between the vagina and the rectum or bladder. One of the common causes in the regions, where obstetric fistulae have a higher incidence, is prolonged obstructed labor [19,20,21]. Obstructed labor causes necrosis of the soft tissues of the pelvis due to ischemia. It results in the opening of an abnormal connection between the vagina and the bladder and/or rectum, with subsequent incontinence of urine and/or feces. The most common obstetric fistula in developing countries is VVF, which involves an abnormal opening between the bladder and the vagina [22]. In Malawi, the estimated prevalence of VVFs is 1.6 in 1000 women [19]. It is estimated that over 2 million women suffer from this condition worldwide, which is consistent with the World Health Organization’s (WHO) current estimates for sub-Saharan Africa and South Asia of between 50,000 to 100,000 cases per year [23]. Uganda has approximately 200,000 women living with fistulae and 2000 annual incident cases, though the numbers may be still underestimated.

The most important risk factors associated with the development of obstetric fistulae are low socioeconomic status, the cultural practice of early marriage of adolescents, inadequate access to and education about family planning, a lack of comprehensive obstetric care, and the long distances from homes to healthcare facilities. Obstetric fistula is a significant problem in low-income countries and is almost non-existent in high-income countries. However, there is evidence suggesting that in many countries, there is an alarming increase in iatrogenic fistulae, and improvement of surgical training and skill-building may likely be needed [24].

Vaginal fistulae have negative consequences for women’s psychological, social, physical, and economic well-being [25]. Patients often face embarrassment, isolation, social stigmatization, and marital separation. They also have a higher risk of developing chronic vaginal and urinary tract infections, renal failure, pelvic inflammatory disease, or amenorrhea [26]. Urine and fecal incontinence may result in an unpleasant odor, skin lesions, malnourishment, amenorrhea, and nerve damage. Women with fistulae are a group at risk of psychological distress and depression. Gladstone et al. aimed to develop an intervention targeting symptoms of depression, anxiety, and traumatic stress in women recovering from fistula repair surgery at the University of Gondar Hospital in Gondar, Ethiopia. Feedback from medical staff members and fistula patients was used to develop a psychological intervention and counseling targeting women recovering from fistula repair surgery [24].

### 3.4. Cesarean Sections and Fistulae

There is an increasing trend in the rate of cesarean sections (CS) worldwide, with even more than 90% of births ending with CS in the private sector in selected countries [27,28,29]. One of the important and severe complications following the increasing number of CS performed is the risk of vesico-uterine fistulae, which represent a rare type of genito-urinary fistulae, representing less than 5% of all urogenital fistulae [30,31]. This kind of abnormal communication between the urinary and genital tracts is reported mostly after CS [32]. Although vesico-uterine fistulae are a rare type of fistulae, there is a risk that the rate of vesico-uterine fistulae will continue augmenting due to the increasing incidence of operative deliveries. This type of fistula can be caused by the presence of a textilloma, consisting of the suture stitches placed when suturing the uterine incision, inadequate mobilization of the urinary bladder during CS, manual extraction of the placenta, instrumental delivery, or pathological insertion of the placenta [2].

Diagnosis may be suggested by the patient’s complaints, such as pelvic pain, amenorrhea, cyclic hematuria—menouria (Youssef syndrome), secondary infertility, or even spontaneous miscarriage [3]. The most common methods used to confirm the diagnosis include intravenous urography, hysteroscopy, cystoscopy, transvaginal ultrasound, or pelvic MRI. Intravenous urography is of low efficacy due to a significant pressure gradient between the urinary tract and uterine corpus. It is one of the most easily performed examinations, which is considered to be a great advantage. Cystoscopy may also help in certain cases with a pathological orifice, indicating an abnormal communication between the urinary bladder and the surrounding organs. MRI remains the mainstay method of imaging vesico-uterine fistulae, with an accuracy rate of up to 100% [8].

Youssef syndrome is defined as the presence of amenorrhea and cyclic hematuria. Patients with an incompetent uterine cervix present with exteriorization of urine through the vagina, while patients in whom the cervix is competent present with hematuria. We can distinguish three types of Youssef syndrome:

- Type I—cyclic menouria without urinary incontinence.
- Type II—urine loss at the vagina level, and some authors also include cases with cyclic menouria, presence of regular menstruation, and urinary incontinence.
- Type III—regular menstruation, and some authors include cases with urinary incontinence, without menouria [3].

A classification of vesico-uterine fistulae according to the routes of menstrual flow was proposed in 2000 by Jóźwik et al. [33].

Conservative treatment is considered an option according to the time of diagnosis and the severity of symptoms. If vesico-uterine fistulae are diagnosed during the early postoperative period, a conservative approach can be proposed. The long evolution of the local inflammatory process leads to a lower chance of self-healing and conservative treatment. When the fistula is diagnosed as a late postoperative complication, conservative treatment is usually not efficient. Then, abnormal communication usually occurs after significant local tissue modifications (such as severe inflammation and fibrosis). Surgery consisting of fistulous trajectory removal en bloc with partial resection and reconstruction of the urinary bladder and the uterine cavity is needed. Surgery can be performed via open, laparoscopic, or robotic approaches, cystoscopy, or hysteroscopy. Then, there is a need for a local reconstruction of the urinary bladder and the uterine cavity [2]. It is to be emphasized that surgical treatment does not exclude the possibility of pregnancy, and spontaneous pregnancies have been reported after such treatment. Minimally invasive surgery and the robotic approach are associated with a significantly better visualization of the structures involved and result in better outcomes. Transvaginal surgery is one of the most modern approaches, still under evaluation, and allows for avoiding an extended intra-abdominal adhesiolysis, though it can be associated with a higher risk of failed closure [34].

Over the years, there have been changes in the distribution of different types of gynecological fistulae. Vesicovaginal fistulae used to be the most common in traditional rural communities, due to the lack of proper hospital and obstetric care and the relatively high incidence of prolonged labors. This situation is still a present problem in the lower-income countries with limited medical care. In the higher-income countries, with a constantly growing rate of cesarean sections and the increasing medicalization of labor, fistulae that are a consequence of medical interventions, including urethra-vaginal and vesico-uterine, have become predominant [14].

A vesico-uterine fistula can be suspected in case of hematuria or urinary incontinence after a CS. Conservative treatment is possible only in cases without urinary incontinence. Surgery, both open and laparoscopic, is the method of choice in the treatment of vesico-uterine fistulae. An operative correction of a fistula should include placing an omental layer between the uterus and the bladder to reduce the risk of fistula recurrence. If there is an indication for a hysterectomy, it should be performed simultaneously with the fistula correction. Among other methods of treatment, the vaginal route and robotic surgery are also recommended, though non-conventional routes should be chosen according to the team’s experience [30]. Surgery should be performed either during the first few days after CS, if there is an early diagnosis, or after two to three months of early puerperium. Early diagnosis is associated with a better treatment prognosis [3].

### 3.5. Pregnancy and Childbirth after Repair of Obstetric Fistula

There are some studies evaluating pregnancy and childbirth in women who underwent repair for obstetric fistula. Delamou et al. indicate that many women in sub-Saharan Africa plan to become pregnant after the repair of their obstetric fistula, despite a high risk of pregnancy complications [22]. Recurrence of the fistula was the most common complication, followed by abortions/miscarriages, stillbirths, and neonatal deaths. Vaginal delivery and emergency CS were associated with an increased risk of stillbirth, recurrence of the fistula, or even maternal death, compared to elective CS. Most of the studies identified elective CS as the main mode of delivery resulting in a better maternal and neonatal outcome of pregnancies after the successful repair of an obstetric fistula. One of the rare types of gynecological fistulae are the cervico-vaginal fistulae, that are in most cases a complication of abortions, but can also can be consequent to a prolonged labor with failed cervical ripening. There are several reported cases of vaginal deliveries through the lacerated cervico-vaginal fistula canal [35].

### 3.6. Schistosoma haematobium and Fistulae

VVF can be caused by *Schistosoma haematobium* infection, as it may result in an impaired wound healing of urogenital tissues. Of the estimated 43 million adults in Sub-Saharan Africa who have symptoms of *S. haematobium* infection, more than 16% suffer from major bladder wall pathology. This may result in an increased rate of obstetric fistulae among women who experience obstructed labor and/or a higher failure rate of fistulae repair. Almost 75% of girls and women with chronic *S. haematobium* infection develop female genital schistosomiasis, a condition in which Schistosoma egg deposition throughout the urogenital tract causes granulomas, sandy lesions, and rubbery papules on the uterus, cervix, and lower genital tract. Considering that the presence of *S. haematobium* in the bladder leads to inflammation, fibrosis, rupture, and ulceration, it can cause a delay in healing after a delivery and possible formation of a fistula. However, Drew et al. indicated that the prevalence of *S. haematobium* was low among patients in an obstetric fistula center in central Malawi and similar to the prevalence of *S. haematobium* in the general population of Malawi [19].

### 3.7. Endometriosis and Fistulae

Endometriosis is a common benign pathological endometrial growth out of the uterine cavity, accompanied by features of chronic inflammation. Most commonly, it affects women of reproductive age, and it is estimated that it affects nearly 10–15% of premenopausal women. The intensity of symptoms is often not related to the extent of the disease, with the most important symptoms of endometriosis being pain of different intensities and localizations, as well as infertility. Deep endometriosis can cause lesions affecting the urinary tract and the intestine, including tenesmus, dyschezia, fecal incontinence, diarrhea, bloating, and dyspareunia [36]. There are different treatment options, from conservative pain-relief medications, through hormone therapy, to surgery, and the optimum treatment method is chosen according to the patient’s symptoms, age, and reproductive plans. Conservative therapy, including contraceptives, intrauterine devices releasing progestagens, or gonadotropin-releasing hormone (GnRH) analogs, is effective only in some patients, associated with numerous side effects, reducing its tolerance, and is limited to patients who do not have reproductive plans in the nearest future. Surgery is the only effective method to achieve long-term, effective treatment of deep endometriosis [37]. There are two surgical approaches: the classical approach, that involves the removal of endometriosis lesions through shaving or excision, and the radical approach, that includes large resections of the intestine. Excision of endometriosis lesions is often associated with postoperative complications, that include the formation of rectovaginal or ureterovaginal fistulae [37]. There are different possible surgical approaches, including transanal, transvaginal, abdominal, and through the perineum [38]. Segmental resection of the intestine is associated with a higher rate of postoperative complications; however, the shaving technique, in the long term, leads to a higher rate of endometriosis recurrence compared to segmental or discoid resection [39].

Intestinal fistulae are among the postoperative complications following surgical techniques performed to treat endometriosis. The CIRENDO study included 364 women with deep endometriosis that infiltrated the rectum. The women were divided according to the technique of the laparoscopic endometriosis procedure they underwent: segmental colon resection (*n* = 139), disc excision (*n* = 80), or rectal shaving (*n* = 145). Postoperative complications were observed in 43 patients (11.8%), including 14 cases of rectovaginal fistulae (3.8%) [40].

A systematic review of English- and French-language articles showed that rectal shaving was associated with a lower rate of postoperative complications than the technique via disc excision or the method of segmental colon resection. However, rectal shaving cannot be used if the endometriotic lesions are infiltrating the colon wall. Excision was associated with a shorter hospital stay and surgery time, as well as a lower risk of intestine stenosis [41].

### 3.8. Crohn’s Disease and Fistulae

Crohn’s disease is a chronic inflammatory disease that most commonly affects the ileum and proximal colon. The inflammation covers the entire width of the intestinal wall. Computed tomography with intravenous contrast or magnetic resonance techniques, such as enterocolitis or enterography, are used to control the stage of the disease, as well as its activity and postoperative recurrences. The first symptoms of the disease are usually anemia and abdominal pain. Majority of symptoms are associated with the digestive system, though the urinary and reproductive tracts can also be involved. Urinary tract complications of Crohn’s disease include urolithiasis, urinary tract infections, or enterovesical fistula [42]. Enterovesical fistula occurs in 2–3.5% of people suffering from Crohn’s disease, and it is the most common cause for enterovesical fistula formation, with a higher prevalence in men than in women. Clinical symptoms indicating the appearance of an enterovesical fistula include fecaluria, pyuria, hematuria, pain, and frequent urinary tract infections, most often of *Escherichia coli* etiology. Enterovesical fistulae can be visualized by oral or rectal administration of the indocyanine green coloring agent [1].

Patients suffering from Crohn’s disease often undergo computed tomography or magnetic resonance imaging; therefore, the radiologist describing the examination should pay attention to abnormalities in the structure of the bladder wall, as well as indirect symptoms such as air in the bladder. Surgical intervention may be the only treatment option for patients with a fistula refractory to conservative treatment [1].

Rectovaginal and ano-vaginal fistulae account for 75% of the gynecological complications of Crohn’s disease. The clinical manifestation is usually vaginal discharge containing air or contaminated with feces, which is often responsible for causing recurrent vaginitis, although there can be cases with no clinical symptoms present. Rectovaginal and ano-vaginal fistulae are most often treated conservatively with the use of biological drugs, and surgery is rarely required. MRI with contrast is used to image fistulae and the progress of their treatment [43].

### 3.9. Vaginoplasty and Fistulae

A typical procedure for transfeminine patients is vaginoplasty [44]. More than 3000 vaginoplasty procedures are performed annually worldwide, making it the most popular operation for gender affirmation. Generally, it is a safe and effective procedure for genital reconstruction in a patient who has undergone gender conversion [45]. The most widely utilized techniques are visceral interposition, pelvic peritoneal vaginoplasty, and penile inversion. Rectovaginal fistulae and vaginal stenosis are uncommon, yet can have significant consequences if present [46]. The precise requirements and surgical objectives of each patient determine the kind of vaginoplasty. If a patient does not expect to ever seek vaginal intercourse or if they have comorbid conditions that make this a safer alternative, they may elect to have a “zero-depth” vaginoplasty.

A rectovaginal fistula is one of the most severe possible complications of transfeminine genital surgery. Fistulae that form without visible rectal damage may be caused by a hidden vascular injury of the rectum and can develop during any postoperative period [46].

Although many fistulae are small in diameter and heal without intervention, fistula repairs may occasionally be required through fecal diversion, fistula resection, or correction of the vaginoplasty. Furthermore, fistulae can appear not only after male-to-female reassignment: urethral fistulae, urethral strictures, and stenosis were also observed in other types of gender-change surgeries, with an incidence of more than 30% [47].

### 3.10. Gynecological Oncology and Fistulae

A total of 1607 patients with advanced-stage epithelial ovarian cancer participated in four randomized controlled trials comparing the effects of neoadjuvant chemotherapy and primary tumor resection surgery. The analysis showed that neoadjuvant chemotherapy was associated with a more optimum cytoreduction, along with a reduction of the rate of perioperative morbidity ans postoperative deaths, and a better QUALY after the surgery. There was also a lower rate of overall complications, including gastrointestinal fistulae (RR: 0.24 (95% CI: 0.06 to 0.95)) [48]. The ICON7 study included 1528 patients with newly diagnosed ovarian cancer who had been randomized into two groups, one that received standard chemotherapy with six cycles of three-weekly intravenous carboplatin and paclitaxel, and the other treated with standard chemotherapy with the addition of bevacizumab. There was a difference in the length of overall survival between patients who received chemotherapy alone (limited mean survival time was 34–35 months (95% CI: 32–37) and patients who received bevacizumab in addition to chemotherapy (limited mean survival time was 39–33 months (95% CI: 37.0–41.7). There were a few cases of serious complications noted among patients treated with bevacizumab, including a case of a gastrointestinal fistula, a case of heart failure, and a case of sarcoidosis [49].

A meta-analysis that compared open abdominal surgery and minimally invasive surgery (MIS) in cervical cancer treatment showed that MIS is associated with a decreased risk of overall intraoperative complications compared to open abdominal surgery postoperative complications (OR: 0.40, 95% CI: 0.34–0.48, *p* < 0.05), including the formation of fistulae [50]. A Chinese study comparing open (*n* = 12,956) and laparoscopic surgery (*n* = 5491) for radical hysterectomy in cervical cancer treatment showed that the laparoscopic approach resulted more often in severe complications, such as intraoperative ureteral damage and postoperative fistulae formation, including ureterovaginal fistula (OR: 4.16, 95% CI: 2.08–8.32), vesicovaginal fistula (OR: 4.16, 95% CI: 2.08–8.32), rectovaginal fistula (OR: 8.04, 95% CI: 1.63–39.53), and ureterovaginal fistula (OR: 4.16, 95% CI: 2.08–8.32) [51].

A study evaluating risk factors for VVFs in patients following accidental cystotomies during elective benign hysterectomies found that women with grade V bladder injuries, according to the American Association for the Surgery of Trauma (AAST), were at a significantly increased risk of formation of VVFs (OR: 93.00, 95% CI: 10.30–838.92) [52]. The study analyzed hysterectomy procedures from two academic centers, performed over a period of eight years. Patients who developed VVF postoperatively were selected and compared with patients who did not develop this complication. In a group of 5698 hysterectomies performed for benign indications, 102 accidental cystotomies were performed, after which 6 patients developed a complication of VVF. The analysis showed that women who went on to develop a VVF underwent statistically longer surgical procedures (317 ± 82 vs. 208 ± 10 min, *p* < 0.05), with a higher incidence of ureteral breaches (33% vs. 1%, *p* < 0.05) and the weight of the uterus usually exceeding 250 g (83% vs. 36%, *p* < 0.05).

## 4. Conclusions

Obstetric fistulae can vary in duration, age, location, severity, size, involvement of the continence mechanism, scarring, and quality of the involved tissues. It is proven that physiotherapy (management of abdominal pressure and pelvic floor training) and health education sessions have a positive influence on the outcome of the surgery, with no adverse effects.

Obstetric fistulae represent a major public health challenge and should be a global healthcare priority during the next decade. Surgery is the only option for many women who are affected by obstetric fistulae. The operation is often difficult because of the anatomical location, the quality of the tissues encountered, involvement of the closure mechanism, and—in some women—previous repair attempts. Most authors agree that the risk of failure increases with an increasing number of previous surgical attempts.

## Data Availability

Not applicable.

## Abbreviations

VVF	vesicovaginal fistula
QUALY	quality of life
RVF	rectovaginal fistula
MRI	Magnetic resonance imaging
FIGO	Fédération Internationale de Gynécologie et d’Obstétrique
ISOFS	International Society of Fistula Surgeons
CS	cesarean section
MIS	minimally invasive surgery
AAST	American Association for the Surgery of Trauma
